# Towards chronic non-invasive stimulation: what can you learn from pain research?

**DOI:** 10.1093/braincomms/fcad193

**Published:** 2023-07-05

**Authors:** Tommaso Bocci, Alberto Priori

**Affiliations:** Clinical Neurology Unit, ASST Santi Paolo & Carlo and Department of Health Sciences, University of Milan, Milan I-20142, Italy; ‘Aldo Ravelli’ Center for Neurotechnology and Experimental Brain Therapeutics, University of Milan, Milan I-20142, Italy; Clinical Neurology Unit, ASST Santi Paolo & Carlo and Department of Health Sciences, University of Milan, Milan I-20142, Italy; ‘Aldo Ravelli’ Center for Neurotechnology and Experimental Brain Therapeutics, University of Milan, Milan I-20142, Italy

## Abstract

This scientific commentary refers to ‘Long-term analgesic effect of trans-spinal direct current stimulation compared to non-invasive motor cortex stimulation in complex regional pain syndrome, by Hodaj *et al*. (https://doi.org/10.1093/braincomms/fcad191).

**This scientific commentary refers to ‘Long-term analgesic effect of trans-spinal direct current stimulation compared to non-invasive motor cortex stimulation in complex regional pain syndrome, by Hodaj *et al*. (https://doi.org/10.1093/braincomms/fcad191).**

Non-invasive brain stimulation techniques, such as transcranial direct current stimulation (tDCS) and repetitive transcranial magnetic stimulation (rTMS), have been used for years to improve symptoms in patients with chronic pain.^1,2^ However, small sample sizes, inappropriate control of experimental conditions and the lack of neurophysiological correlates underlying the clinical outcome still limit a more extensive use of these techniques in the clinical practice. Moreover, most papers adopt classical protocols lasting for 1 or 2 weeks but little is known about the effects of longer treatments. Another limitation is the lack of studies comparing different neuromodulation techniques, both invasive and non-invasive.

In the paper by Hodaj and co-workers,^3^ the authors compared three different neuromodulation techniques, spinal cord stimulation (SCS), repetitive transcranial magnetic stimulation (rTMS) and transcranial direct current stimulation (tDCS); moreover, for patients treated with tDCS, they proposed an innovative protocol, in which each patient underwent a series of 12 sessions of stimulation for 3 weeks (namely, the ‘induction phase’), followed by 11 sessions for 4 months (the ‘maintenance therapy’). The impact on the future of NIBS may be strong: the opportunity to use weak currents for longer periods may lead to the best clinical outcome, possibly interfering with gene expression, pro-inflammatory response and cortical somatotopic reorganization. The term ‘electroceutics’ is now emerging and defines the possibility to modifying disease progression over time, not only in chronic pain syndromes but also in neurodegenerative disorders.^4^ Also, growing evidence in pre-clinical models strongly supports this hypothesis.^5^ To the best of our knowledge, this is the first paper pointing to a ‘chronic’ use of tDCS, possibly applying electric fields weaker than those used before.

Another key point is represented by the stronger effect observed for transcutaneous spinal direct current stimulation (tsDCS) when compared to repetitive transcranial magnetic stimulation and spinal cord stimulation. The authors explain these results by assuming that non-invasive spinal stimulation may interfere with supra-spinal pathways. That is confirmed by data in healthy subjects showing that transcutaneous spinal direct current stimulation is able to modulate intra-cortical excitability, inter-hemispheric transfer and motor recruitment.^6^ The opportunity to modulate at different levels nociceptive processing is of key importance, especially because both chronic pain and the so-called ‘central sensitization’ (a pain experience not linearly correlated with the entity of nociceptive stimuli) arise from a combination of dysfunctional networks, including a cortical reorganization of sensorimotor maps, a phenotypic switch in the expression of spinal neuropeptides and the possible co-existence of an autonomic dysreflexia (Fig. 1).^7,8^ In this connection, central sensitization still remains a challenge, both for clinicians and neuroscientists, and represents the main hurdle to develop effective therapies for the treatment of refractory pain syndromes, involving different dysfunctional levels and a vast number of networks and neurotransmitters. Along this way, gene therapy has been recently proposed for pain treatment in mice but its use is highly debated in humans.^9^

**Figure 1:**
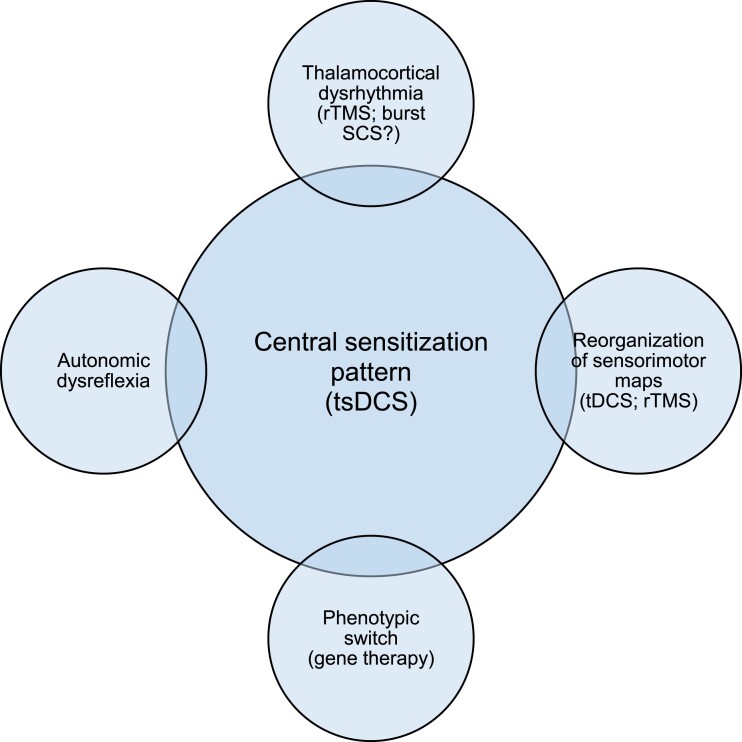
**Pathophysiological mechanisms underlying central sensitization in pain syndromes.** Central sensitization still represents a challenge for clinicians, and its mechanisms have been only partly elucidated, comprising both bottom-up and top-down pathways. Some of these mechanisms may be modulated either by invasive [spinal cord stimulation (SCS)] or by non-invasive treatments (tDCS, transcutaneous spinal direct current stimulation and repetitive transcranial magnetic stimulation), with transcutaneous spinal direct current stimulation showing promising results due the possibility to interfere both with spinal and with supra-spinal circuitries.

Also, spinal cord stimulation has been shown to interfere with supra-spinal nociceptive processing and cortical networks, especially when theta-burst stimulation (TBS) is delivered,^10,11^ but its clinical impact is weak for some clinical syndromes which represent a paradigm of chronic pain, such as phantom limb pain (PLP).

The paper by Hodaj and colleagues^3^ may help to elucidate many of these aspects, towards a novel conceptualization of pain treatment and non-invasive brain stimulation techniques, in the field of neuroprotection and long-term treatments.
